# The basophil activation test differentiates between patients with wheat‐dependent exercise‐induced anaphylaxis and control subjects using gluten and isolated gluten protein types

**DOI:** 10.1002/clt2.12050

**Published:** 2021-08-05

**Authors:** Angelika Miriam Gabler, Julia Gebhard, Bernadette Eberlein, Tilo Biedermann, Katharina Anne Scherf, Knut Brockow

**Affiliations:** ^1^ Leibniz‐Institute for Food Systems Biology at the Technical University of Munich Freising Germany; ^2^ Department of Dermatology and Allergy Biederstein TUM School of Medicine Technical University of Munich Munich Germany; ^3^ Department of Bioactive and Functional Food Chemistry Institute of Applied Biosciences Karlsruhe Institute of Technology (KIT) Karlsruhe Germany

**Keywords:** ω5‐gliadin, basophil activation test, gluten, wheat allergy, wheat‐dependent exercise‐induced anaphylaxis (WDEIA)

## Abstract

**Background:**

Oral food challenge using gluten and cofactors is the gold standard to diagnose wheat‐dependent exercise‐induced anaphylaxis (WDEIA), but this procedure puts patients at risk of an anaphylactic reaction. Specific IgE to ω5‐gliadins as major allergens and skin prick tests to wheat may yield negative results. Thus, we designed a proof‐of‐principle study to investigate the utility of the basophil activation test (BAT) for WDEIA diagnosis.

**Methods:**

Different gluten protein types (GPT; α‐, γ‐, ω1,2‐ and ω5‐gliadins, high‐molecular‐weight glutenin subunits [HMW‐GS] and low‐molecular‐weight glutenin subunits [LMW‐GS]) and gluten were used in different concentrations to measure basophil activation in 12 challenge‐confirmed WDEIA patients and 10 control subjects. The results were compared to routine allergy diagnostics. Parameters analyzed include the percentage of CD63^+^ basophils, the ratio of %CD63^+^ basophils induced by GPT/gluten to %CD63^+^ basophils induced by anti‐FcεRI antibody, area under the dose‐response curve and test sensitivity and specificity.

**Results:**

GPT and gluten induced strong basophil activation for %CD63^+^ basophils and for %CD63^+^/anti‐FcɛRI ratio in a dose‐dependent manner in patients, but not in controls (*p* < 0.001, respectively). BAT performance differed from acceptable (0.73 for LMW‐GS) to excellent (0.91 for ω5‐gliadins) depending on the specific GPT as evaluated by the area under the receiver operating characteristic curve. Patients showed individual sensitization profiles. After determination of the best cut‐off points, ω5‐gliadins and HMW‐GS showed the best discrimination between patients and controls with a sensitivity/specificity of 100/70 and 75/100, respectively.

**Conclusion:**

This study shows the alternative role of BAT in better defining WDEIA and the causative wheat allergens. The best BAT parameters to distinguish WDEIA patients from controls were %CD63^+^ basophil values for ω5‐gliadins and HMW‐GS.

## 1 INTRODUCTION

Wheat‐dependent exercise‐induced anaphylaxis (WDEIA) is a rare, but potentially life‐threatening cofactor‐induced wheat allergy. ω5‐gliadins and high‐molecular‐weight glutenin subunits (HMW‐GS) are most often reported as major allergens, but reactions to other gluten protein types (GPTs) from wheat, like low‐molecular‐weight glutenin subunits (LMW‐GS), α‐ and γ‐gliadins were also described. All GPT together constitute gluten, the storage proteins in wheat flour.^1^, ^2^, ^3^, ^4^

WDEIA diagnosis is challenging, because of the variety of possible allergenic wheat proteins and the combination with a cofactor. Skin prick tests (SPTs) and specific IgE (sIgE) to wheat may be negative. Even sIgE to ω5‐gliadins are only positive in about 80% of WDEIA patients, indicating that other GPT also play a role in WDEIA. Wheat product and exercise challenge failed to induce symptoms in the majority of patients despite a clear history.^1^, ^4^, ^5^ Thus, oral food challenge with gluten, that has a protein content of 70%–80% compared to the 8%–15% in wheat flour, combined with cofactors is often needed to overcome non‐responsiveness.^6^, ^7^

A new approach to complement WDEIA diagnosis is the basophil activation test (BAT) combined with florescence‐activated cell sorting. Stimulation with an allergen‐containing solution (allergen test solution [ATS]) induces upregulation of the expression of cell surface proteins, such as CD63 or CD203c. BAT‐derived parameters such as the percentage of basophils that respond to a given dose of the ATS or the area under the curve (AUC) of a dose‐response curve have been shown to be sensitive biomarkers corresponding to the clinical severity of anaphylactic reactions.^8^, ^9^ The BAT has been established for the identification of different immediate allergies, like allergy against wheat,^8^, ^10^ hymenoptera venom,^11^ and alpha‐galactose.^12^

The aim of this proof‐of‐principle study was to investigate the utility of the BAT to improve WDEIA diagnosis and to determine individual sensitization profiles in WDEIA patients to different isolated and well‐characterized GPT and gluten.

## 2 METHODS

### 2.1 Study population

The following exclusion criteria were considered in the selection of participants to avoid potential confounding factors and/or health risk to any of the participants: Pregnancy/lactation; systemic intake of corticosteroids (cortisone) 3 weeks and/or antihistamines (anti‐pruritic drugs) 1 week before the start of the test; intake of laxatives, anti‐diarrhea drugs, thyroid hormone preparations, antibiotics, immunosuppressive drugs, analgesic drugs (aspirin, NSAIDs, etc.) taking psychotropic drugs and certain blood pressure medications (ACE inhibitors, ß‐blockers); serious internal diseases (gastrointestinal, neurological, cardiovascular, rheumatic diseases, celiac disease, cancer, kidney diseases, acute infections, etc.); bronchial asthma.

A total of 23 participants were consecutively recruited from the medical center (15 f, 8 m, 25–76 years). Twelve of them were patients with a history of WDEIA based on positive oral food challenge, SPT, sIgE, and clinical history (5 f, 7 m, 26–60 years, Table 1). Provocations had been done with 8–32 g gluten intake as described.^7^, ^13^ Some patients, depending on their history, were given increasing doses of cofactors (500–1000 mg of ASA ± 10–20 ml of 95% ethanol; Braun, Melsungen, Germany diluted with 200 ml of black currant‐flavored water) 30 min before gluten challenge and standardized aerobic and anaerobic exercise was undertaken 30–60 min after gluten ingestion. Eleven individuals without a history of any wheat‐related disorder were included in the study as controls (10 f, 1 m, 25–76 years, Table 2).

**TABLE 1:** Characteristics of WDEIA patients, diagnosed by oral food challenge: sex, age, BMI, atopic dermatitis, total IgE (KU/L), sIgE (KU/L) against wheat flour, rye flour, gluten, gliadins, ω5‐gliadin, lipid‐transfer protein, *dermatophagoides pteronyssinus*, timothy grass, birch pollen allergen, cofactor, symptoms, severity according to Messmer and Ring and involved organ systems

Patients	Sex	Age	BMI	AD	Total IgE	sIgE									Cofactor	Symptoms	Severity
						WF	RF	G	Glia	ω5	Tri a 14	DP	TG	Bet v1			
1	F	45	19	‐	203.0	0.72	0.66	0.65	0.29	8.12	<0.10	0.28	0.37	<0.10	Exercise, NSAID, stress, mental strain	i, f, u, g, gi, v	II (skin, gut)
2	M	39	21	‐	97.3	0.32	0.73	0.80	1.10	3.47	<0.10	0.34	<0.10	<0.10	Exercise	i, f, u, g	I (skin)
3	M	42	28	RCA, A	105.0	0.83	0.82	0.73	0.87	3.97	<0.10	<0.10	0.82	<0.10	Exercise, stress, mental strain	i, u, r, un, d	III (skin, respiratory tract, CVS)
4	M	60	32	RCA	50.0	0.47	1.19	2.08	1.53	4.98	<0.10	<0.10	<0.10	0.32	Exercise, stress	i, u, un	III (skin, CVS)
5	M	48	29	‐	95.0	0.32	0.26	0.28	0.13	1.52	<0.10	0.22	<0.10	<0.10	Exercise	i, f, un	III (skin, CVS)
6	M	54	24	RCA, A	319.0	1.44	3.40	5.89	4.67	10.70	<0.10	0.17	0.60	<0.10	Exercise, NSAID, stress, cold, heat, mental strain	i, u, h	II (skin, CVS)
7	F	47	37	‐	95.3	0.14	0.16	0.22	<0.10	0.32	<0.10	0.25	<0.10	<0.10	Exercise, NSAID, alcohol	u, r, un	III (skin, respiratory tract, CVS)
8	M	55	27	RCA	368.0	2.70	2.98	7.38	5.10	11.40	<0.10	0.9	2.49	5.15	Exercise, alcohol, cold, heat	i, f, u, un	III (skin, CVS)
9	F	52	23	‐	172.0	0.55	1.51	2.41	2.25	5.04	<0.10	<0.10	3.55	0.75	Exercise, AA, alcohol	i, f, u, r, g, un, h	III (skin, respiratory tract, CVS)
10	F	54	29	‐	90.3	0.47	1.14	1.26	0.64	4.09	<0.10	<0.10	0.17	<0.10	AA, stress, cold, mental strain	i, f, u	I (skin)
11	M	56	29	RCA, AE	2193.0	5.95	7.04	11.70	11.00	30.20	<0.10	15.60	>100	<0.10	AA	i, f, u, r, g, v	II (skin, gut)
12	F	26	23	‐	82.6	0.21	0.45	<0.10	<0.10	<0.10	<0.10	0.38	1.46	0.20	Exercise	i, f, u, g	I (skin)

Abbreviations: A, asthma; AA, acetylsalicylic acid; AD, atopic disease; AE, atopic eczema; Bet v1, birch pollen allergen; BMI, body mass index; CVS, cardiovascular system; DP, *dermatophagoides pteronyssinus*; f, flush; g, globus sensation; G, gluten; gi, gastrointestinal complaints; Glia, gliadins; h, hypotension; i, itching; NSAID, non‐steroidal anti‐inflammatory drugs; OAS, oral allergy syndrome; r, respiratory distress; RCA, allergic rhinoconjuctivitis; RF, rye flour; sIgE, specific immunoglobulins; TG, timothy grass; Tri a 14, lipid‐transfer protein; u, urticaria; un, unconsciousness; v, vomiting; WF, wheat flour; ω5, ω5‐gliadin.

**TABLE 2:** Characteristics of controls: sex, age, atopic dermatitis, total IgE (KU/L), sIgE (KU/L) against wheat flour, rye flour, gluten, gliadins, ω5‐gliadin, lipid‐transfer protein, *dermatophagoides pteronyssinus*, timothy grass, birch pollen allergen

Controls	Sex	Age	AD	Total IgE	sIgE								
					WF	RF	G	Glia	ω5	Tri a 14	DP	TG	Bet v1
1	F	76	‐	82.7	<0.10	<0.10	<0.10	<0.10	<0.10	<0.10	<0.10	<0.10	<0.10
2	F	71	RCA, OAS	38.9	<0.10	<0.10	<0.10	<0.10	<0.10	<0.10	<0.10	<0.10	15.9
3	F	27	RCA, OAS	44.2	<0.10	<0.10	<0.10	<0.10	<0.10	<0.10	<0.10	6.16	5.30
4	F	26	‐	6.7	<0.10	<0.10	<0.10	<0.10	<0.10	<0.10	<0.10	<0.10	<0.10
5	F	25	‐	5.7	<0.10	<0.10	<0.10	<0.10	<0.10	<0.10	<0.10	<0.10	<0.10
6	F	32	RCA, A	49.8	<0.10	<0.10	<0.10	<0.10	<0.10	<0.10	2.62	0.86	1.72
7	F	26	‐	15.0	<0.10	<0.10	<0.10	<0.10	<0.10	<0.10	<0.10	<0.10	<0.10
8	F	51	‐	3.5	<0.10	<0.10	<0.10	<0.10	<0.10	<0.10	<0.10	<0.10	<0.10
9	M	54	RCA	28.0	<0.10	<0.10	<0.10	<0.10	<0.10	<0.10	2.87	1.86	<0.10
10	F	55	RCA	960.0	1.95	1.68	0.19	<0.10	<0.10	<0.10	0.33	2.36	>100

Abbreviations: A, asthma; AE, atopic eczema; AD, atopic disease; Bet v1, birch pollen allergen; DP, *dermatophagoides pteronyssinus*; G, gluten; Glia, gliadins; OAS, oral allergy syndrome; RCA, allergic rhinoconjuctivitis; RF, rye flour; sIgE, specific immunoglobulins; TG, timothy grass; Tri a 14, lipid‐transfer protein; ω5, ω5‐gliadin; WF, wheat flour.

The study protocol was approved by the ethics committee of the Technical University of Munich and all participants gave written informed consent before being included in the study.

### 2.2 Skin prick test

SPT was carried out on the forearm with the following substances: wheat flour, gluten, isolated LMW‐GS, HMW‐GS, and gliadins. A 10% histamine‐dihydrochloride solution (ALK‐Abello, Hørsholm, Denmark) was used as positive and isotonic sodium chloride solution (Fresenius Kabi Deutschland GmbH) as negative control.^7^ Details of the production and characterization of GPT can be found in the Supporting Methods.

### 2.3 Serum sIgE and total IgE

Serum sIgE and total IgE levels were measured by ImmunoCap and Phadia 250 (Thermo Fisher Scientific). Serum sIgE levels of the following allergens were determined: *Dermatophagoides pteronyssinus* (d1), timothy grass (g6), birch pollen allergen (Bet v 1, t215), wheat flour (f4), rye flour (f5), gluten (f79), gliadin (f98), ω5‐gliadin (Tri a 19, f416), and lipid‐transfer protein (Tri a14, f433).

### 2.4 Preparation of BAT ATSs

Gliadins were extracted from wheat gluten using 60% aqueous ethanol. After dialysis and lyophilization, the gliadin fraction was separated into ω5‐, ω1,2‐, α‐, and γ‐gliadins by preparative reversed‐phase high‐performance liquid‐chromatography. The glutenins were extracted from the residue after gliadin removal using 50% aqueous propanol, 60°C and reducing conditions. The HMW‐GS and LMW‐GS were obtained by sequential precipitation with 40% and 80% acetone, respectively.^14^, ^15^, ^16^ Details of the production and characterization of the GPT can be found in the Supporting Methods.

GPT or gluten (15 mg) and 0.6 ml pepsin solution (0.6 mg/ml pepsin solved in 0.01 mol/L hydrochloric acid, enzyme‐substrate ratio of 1/25) were incubated for 120 min at 37°C. The digest was stopped by adjusting the pH value to 7.0 with sodium hydrogen carbonate solution (50 mg/ml). The solution was filtered (0.45 μm) and the protein/peptide concentrations were measured at 205 nm by a micro volume UV/VIS spectrophotometer NanoDrop One (Thermo Fisher Scientific). If necessary, the sample solution was diluted with water to a concentration of 4 mg/ml. Further dilutions were made, to receive the following concentrations: 2.0, 0.8, 0.4, and 0.08 mg/ml. A pepsin‐control was prepared in the same way, but without gluten proteins. ATS were prepared and stored at −20°C in aliquots until use in BAT.

### 2.5 Basophil activation test

For quantitative determination of in vitro basophil activation, Flow CAST (Buehlmann Laboratories AG) was used, as described previously.^12^ Venous blood was collected from participants in EDTA tubes and used immediately. The blood samples were gently homogenized at room temperature (RT). Per measurement, 50 μl of ATS (concentration 4.0–0.2 mg/ml), 100 μl stimulation buffer, 50 μl blood and 20 μl staining reagent were gently mixed by hand in polystyrene tubes. The staining reagent consisted of anti‐CD63‐fluorescein‐isothiocyanate and anti‐CCR3‐pycoerythrin monoclonal antibodies (mAb). The tubes were then incubated for 25 min at 37°C. By addition of 2 ml lysis reagent and standing for 5 min in the dark at RT, the stimulation was stopped. The tubes were centrifuged at 500 × *g* for 5 min. The supernatant was decanted and the residue was resuspended in 200 μl of wash buffer by gentle mixing. Highly specific anti‐FcεRI mAb and N‐formyl‐methionyl‐leucyl‐phenylalanine were used as positive controls. To determine the background value, stimulation buffer alone was used. The flow cytometric analysis was performed using a FACSCalibur system (Becton‐Dickinson Immunocytometry System) with a 488 nm, 15 mW and a 635 nm, 10 mW argon laser. Basophils were gated as low side scatter CCR3/side scatter^low^. CCR3 was used to identify basophils and CD63 as basophil activation marker, both marked with fluorescence‐dye‐labeled mAb. BD CellQuest (Becton‐Dickinson Immunocytometry System) was used for data analysis. In each measurement, ≥450 basophil granulocytes (BG) were counted. The upregulation of the basophil activation marker CD63 by the tested ATS reflects the induced basophil activation.^11^, ^12^, ^17^, ^18^

### 2.6 Determination of different BAT parameters

The basophil activation (%CD63^+^ basophils) was calculated by the percentage of CD63‐expressing BG relative to the total number of counted BG in each measurement. The %CD63^+^ basophils/anti‐FcεRI ratio is defined as the quotient of the maximum percentage of activated %CD63^+^ basophils, induced by an IgE‐dependent stimulus, and the percentage of activated basophils triggered by the anti‐FcεRI mAb as positive control.

### 2.7 Statistical analysis

Statistical analysis was performed with SigmaPlot 14 (Systat Software GmbH) and Origin 19 (OriginLab Corporation). Statistical significance was tested by one‐way ANOVA and Dunn's post hoc test. Receiver operating characteristic (ROC) analyses were carried out to estimate the discriminatory ability of the investigated parameters. Therefore, the area under the ROC curve, was used as further characteristic. The optimized cut‐off of basophil activation (%) for best selectivity and specificity was determined from the ROC curve. Correlations between BAT results (maximum %CD63^+^ basophils, %CD63^+^/FcεRI ratio), diameter of wheals and erythema in SPT, severity (grouped comparisons I, II, III) and sIgE values were analyzed using Spearman's correlation test.

## 3 RESULTS

### 3.1 Study population

Twelve patients (7 m, 5 f; age range: 26–60 years; median age: 48 years) with a clinical history of WDEIA and positive challenge test were included in the study (Table 1). The control population consisted of 11 controls without a clinical history of any wheat‐related disorder; six subjects were atopic. One subject with atopy was excluded, because of non‐responsiveness to the positive control anti‐FcɛRI mAb in the BAT. Therefore, 10 controls were analyzed further (1 m, 9 f; age range: 25–76 years; median age: 44 years) (Table 2).

### 3.2 SPT, serum sIgE and total IgE

**FIGURE 1:**
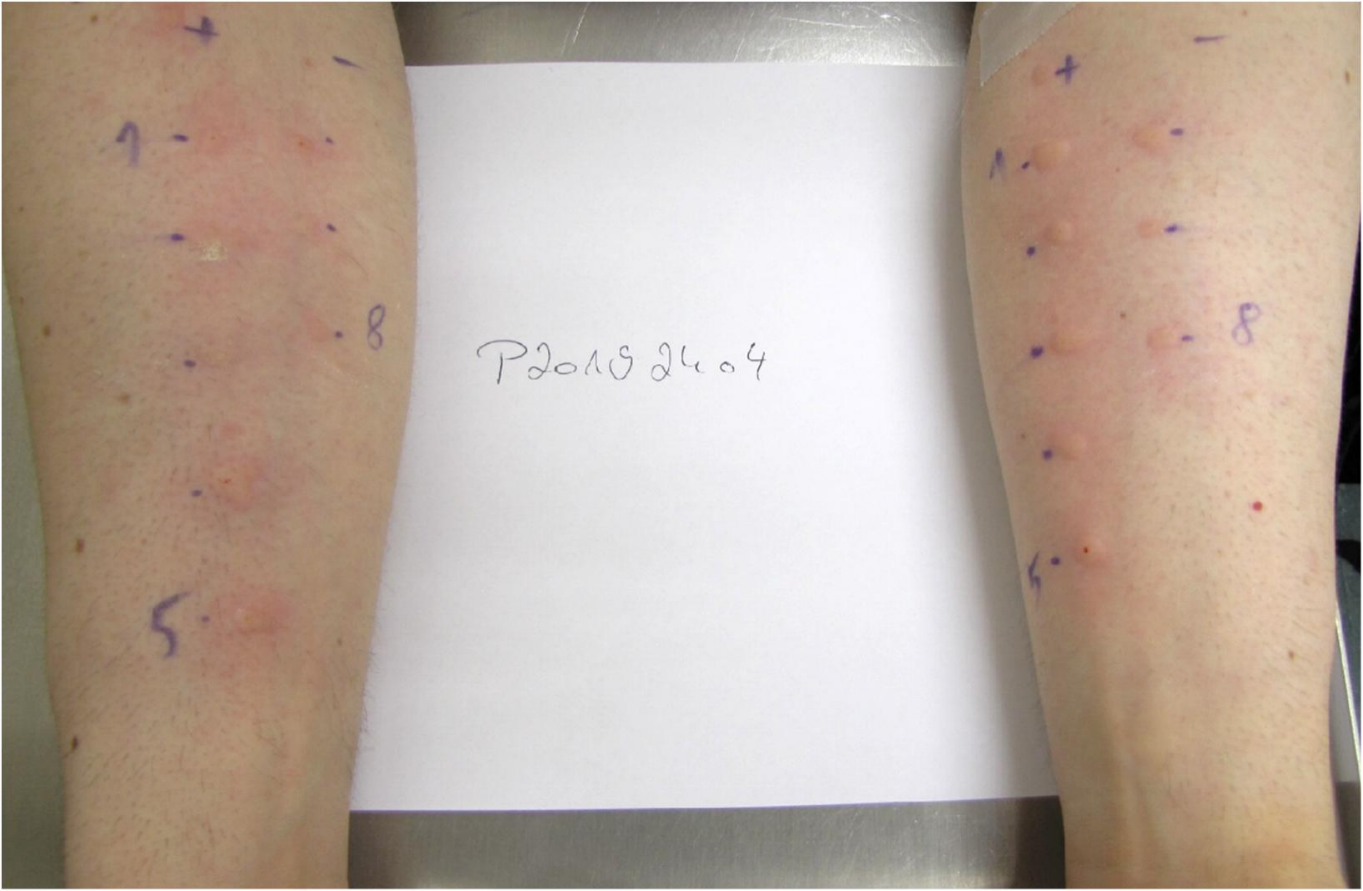
Exemplary skin prick test (SPT) results for wheat‐dependent exercise‐induced anaphylaxis patient 3. The following SPT substances were applied: +: histamine positive control, −: sodium chloride negative control, 1: gluten, 2: strongly hydrolyzed wheat protein, 3: slightly hydrolyzed wheat protein, 4: low‐molecular‐weight glutenin subunits, 5: high‐molecular‐weight glutenin subunits, 6: gliadins, 7: special gluten sample (significantly lower ω5‐gliadin content), 8: wheat flour

A SPT is classified as positive when the diameter of the wheal, caused by the test substance, is greater or equal than the diameter of the wheal of the negative control with 3 mm added. Patients did not show wheals for the negative control, and they all showed a distinct allergic reaction to the positive control. There were positive responses to gluten and gliadins in SPT in all patients (Table 3, Figure 1). In case of wheat flour, HMW‐GS and LMW‐GS positive results were obtained with only two exceptions. Patients p7 and p12 were the only ones showing negative results to some of the test substances (p7: wheat flour, p12: wheat flour, LMW‐GS, HMW‐GS). There were no significant differences in wheal or erythema diameter between wheat flour, gluten, gliadins, HMW‐GS, and LMW‐GS between the patients (*p* > 0.05).

**TABLE 3:** Skin prick test results for WDEIA patients (p) to wheat flour, gluten, gliadins and HMW‐GS/LMW‐GS

Patients	Wheat flour		Gluten		Gliadins		LMW‐GS		HMW‐GS		Histamine		NaCl	
	W	E	W	E	W	E	W	E	W	E	W	E	W	E
1	**4.0**	13.0	**6.5**	14.0	**7.0**	18.0	**4.5**	9.0	**4.5**	11.0	6.0	8.0	0.0	0.0
2	**10.5**	15.0	**9.0**	15.0	**7.0**	10.0	**9.0**	16.0	**12.5**	18.0	6.0	9.0	0.0	2.0
3	**6.5**	9.0	**6.0**	17.0	**7.0**	15.0	**7.5**	18.0	**7.0**	17.0	7.0	10.0	0.0	0.0
4	**6.0**	7.0	**7.0**	10.0	**7.0**	8.0	**10.0**	15.0	**10.0**	13.0	6.0	8.0	0.0	0.0
5	**6.0**	9.0	**4.0**	14.0	**4.0**	5.0	**7.0**	20.0	**5.0**	20.0	6.0	8.0	0.0	2.0
6	**6.0**	15.0	**6.0**	14.0	**4.5**	14.0	**9.0**	23.0	**7.0**	19.0	6.0	13.0	0.0	0.0
7	2.5	5.0	**5.5**	11.0	**3.0**	5.0	**4.5**	9.0	**5.5**	8.0	6.0	11.0	0.0	0.0
8	**7.0**	18.0	**12.0**	31.0	**8.5**	24.0	**12.0**	24.0	**6.0**	17.0	6.0	19.0	0.0	2.0
9	**5.0**	18.0	**3.5**	10.0	**7.5**	15.0	**3.0**	9.0	**5.0**	16.0	6.0	21.0	0.0	0.0
10	**5.5**	22.0	**4.4**	21.0	**5.0**	23.0	**5.5**	25.0	**5.5**	22.0	4.0	15.0	0.0	0.0
11	**8.0**	18.0	**6.5**	21.0	**12.0**	27.0	**11.0**	28.0	**8.5**	25.0	6.0	21.0	0.0	2.0
12	1.0	2.0	**3.0**	7.0	**3.5**	6.0	1.5	2.0	2.5	4.0	5.0	18.0	0.0	1.0
Range	1.0–12.0	2.0–25.0	3.0–15.0	5.0–35.0	3.0–11.0	5.0–32.0	1.0–18.0	1.0–30.0	2.0–13.0	3.0–30.0	3.0–7.0	7.0–30.0	‐	0.0–2.0
Median	5.8	12.6	6.1	15.4	6.3	14.2	7.0	16.5	6.6	15.8	5.8	13.4	0.0	0.8

*Note:* The results are the median of a double determination *n* = 2 (except WDEIA patient 4, *n* = 1). Isotonic sodium chloride was used as negative (NaCl) and a 10% histamine solution as positive control (histamine). The diameters for wheals and erythema were documented in mm. A result is classified as positive (marked in bold), when the diameter of the wheal, caused by a test substance, is greater or equal than the diameter of the wheal caused by the negative control with 3 mm added. The range and median of all patients' results per test substance is documented.

Abbreviations: E, erythema; HMW, high‐molecular weight glutenin subunits; LMW‐GS, low‐molecular weight glutenin subunits; W, wheals; WDEIA, wheat‐dependent exercise‐induced anaphylaxis.

Significantly higher values were found for total IgE and sIgE against *Dermatophagoides pteronyssinus*, timothy grass, wheat flour, rye flour, gluten gliadins, and ω5‐gliadins in patients compared to controls (*p* < 0.05), respectively. No significant differences between patients and controls were found for sIgE against birch pollen allergen (Bet v 1) and lipid‐transfer protein (Tri a14) (*p* > 0.05). For details see Tables 1 and 2.

### 3.3 Evaluation of the response induced by ATSs in BAT

**FIGURE 2:**
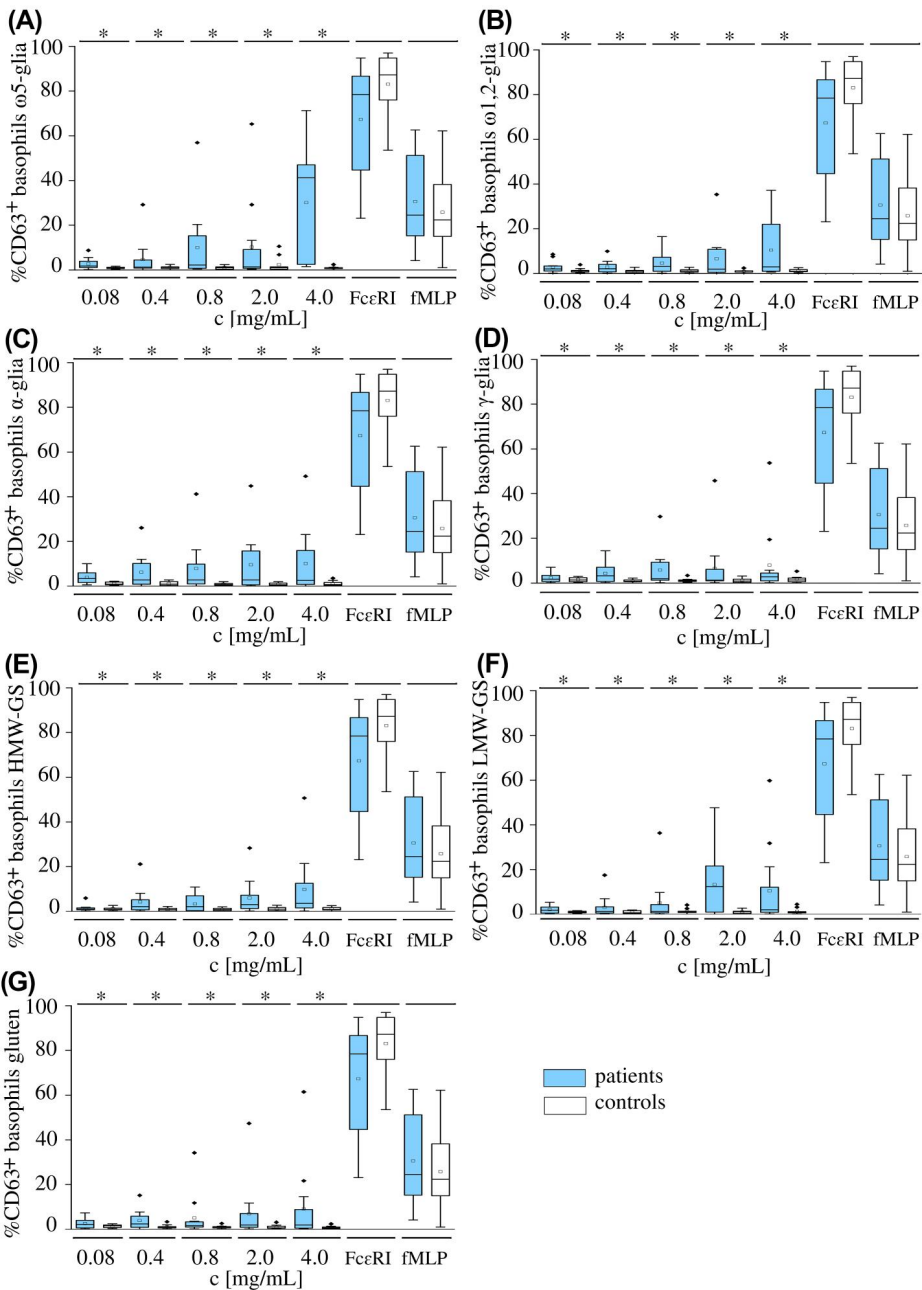
Dose‐dependent basophil activation (%CD63^+^ basophils) in wheat‐dependent exercise‐induced anaphylaxis patients (blue) and controls (white) using allergen test solutions from gluten protein types ω5‐gliadins (A), ω1,2‐gliadins (B), α‐gliadins (C) and γ‐gliadins (D), high‐molecular‐weight glutenin subunits (E) and low‐molecular‐weight glutenin subunits (F), and gluten (G) at concentrations of 4.0, 2.0, 0.8, 0.4, and 0.08 mg/ml and the positive controls anti‐FcɛRI monoclonal antibody and N‐formyl‐methionine‐leucyl‐phenylalanine (fMLP). Significant differences between patients and controls are indicated by asterisks (one‐way ANOVA, Dunn's post hoc test, *p* < 0.001). Diamonds indicate individual outliers beyond the interquartile range

The induced allergenic response to the ATS was evaluated by three parameters used in BAT: %CD63^+^ basophils, %CD63^+^/anti‐FcɛRI ratio and AUC of dose‐response curves. The ATS made from gluten and GPT induced basophil activations in patients with WDEIA. The basophil activation was dose‐dependent up to a maximum of 71.3 %CD63^+^ basophils in case of ω5‐gliadins, 61.5% for gluten, 59.8% for LMW‐GS, 53.7% for γ‐gliadins, 50.7% for HMW‐GS, 49.2% for α‐gliadins, and 37.3% for ω1,2‐gliadins. Significant differences between patients and controls were found for each ATS at every concentration tested (*p* < 0.001) (Figure 2). There were no significant differences in the background values for patients (range: 0.4%–1.7% CD63^+^ basophils, median: 0.9% CD63^+^ basophils) and controls (range: 0.4%–3.1% CD63^+^ basophils, median: 1.1% CD63^+^ basophils). The %CD63^+^/anti‐FcɛRI ratio was significantly higher for gluten and all GPT for patients compared with controls at most concentrations (*p* < 0.001) (Figure S1). The dose‐response curves were generated from the values of %CD63^+^ basophils. The AUC as evaluation parameter combines the triggered allergic response (%CD63^+^ basophils) and all tested doses in one. The AUC of ω5‐gliadins, α‐gliadins, and HMW‐GS were significantly higher in patients compared to controls (*p* < 0.001), but there were no significant differences for ω1,2‐gliadins, γ‐gliadins, LMW‐GS, and gluten (*p* > 0.05). For patients, median AUC values were 56.5 (range: 3.8–232.2) for ω5‐gliadins, 34.7 (range: 0.4–163.7) for α‐gliadins, and 24.0 (range: 3.7–102.9) for HMW‐GS. Table 4 shows the AUCs of dose‐response curves of gluten and GPT for patients and controls.

**TABLE 4:** Area under the dose‐response curve (%CD63^+^ basophils by concentration of the allergen test solutions [mg/ml]) from patients and controls for gluten and ω5‐, ω1,2‐, α‐, and γ‐gliadins and HMW‐GS/LMW‐GS

	Gluten	ω5‐gliadins	ω1,2‐gliadins	α‐gliadins	γ‐gliadins	HMW‐GS	LMW‐GS
p1	44.4	114.2	44.0	54.4	10.6	28.0	46.9
p2	12.6	4.9	24.9	39.4	18.7	35.2	41.1
p3	2.8	3.8	4.2	7.9	7.3	17.2	40.7
p4	5.4	7.0	6.6	8.4	9.0	19.0	20.5
p5	2.8	6.5	2.6	2.6	3.7	3.7	3.1
p6	44.8	80.8	48.7	63.8	49.0	49.7	80.7
p7	1.7	6.5	3.0	0.4	0.8	0.9	2.3
p8	3.0	44.3	1.1	1.9	3.3	1.3	0.5
p9	170.4	232.2	108.1	163.7	156.2	102.9	172.1
p10	9.4	50.7	3.1	3.7	4.0	8.4	4.3
p11	9.8	67.2	12.4	11.8	7.9	8.7	9.3
p12	7.5	59.8	56.6	58.1	29.9	12.9	24.6
Range (p)	1.7–170.4	3.8–232.2	1.1–108.1	0.4–163.7	0.8–156.2	0.9–102.9	0.5–172.1
Median (p)	26.2	56.5	26.3	34.7	25.0	24.0	37.2
c1	3.6	3.8	2.1	1.3	2.5	1.7	2.4
c2	3.8	3.3	1.5	6.9	8.1	1.9	6.5
c3	6.3	3.8	5.9	6.2	6.6	8.4	6.7
c4	4.4	4.2	4.7	4.4	5.0	7.2	5.2
c5	2.1	2.2	3.4	1.4	3.0	4.1	3.8
c6	9.8	16.3	9.9	8.3	11.2	7.4	9.4
c7	3.3	3.9	5.4	3.7	5.0	4.4	6.3
c8	1.6	2.7	2.3	2.2	3.5	0.6	1.5
c9	3.1	18.3	2.8	1.8	5.8	4.6	3.7
c10	1.4	2.0	3.3	1.5	1.2	1.5	1.6
Range (c)	1.4–9.8	2.0–18.3	1.5–9.9	1.3–8.3	1.2–11.2	0.6–8.4	1.5–9.4
Median (p)	3.9	6.1	4.1	3.8	5.2	4.2	4.7

Abbreviations: c, control; HMW‐GS, high‐molecular‐weight glutenin subunits; LMW‐GS, low‐molecular‐weight glutenin subunits; p, patient.

### 3.4 ROC curves

**FIGURE 3:**
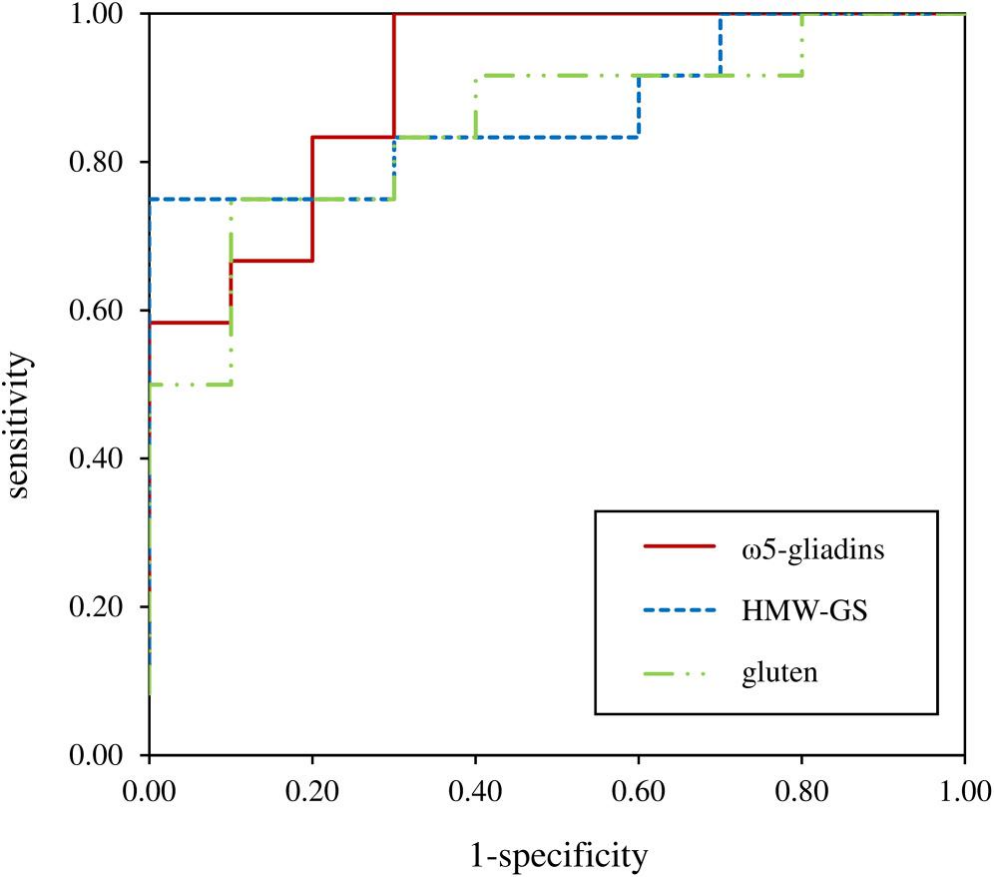
Concentration‐independent receiver operating characteristic curves for ω5‐gliadins, high‐molecular‐weight glutenin subunits (HMW‐GS), and gluten, which had the highest sensitivity and specificity. The maximum basophil activation %CD63^+^ basophils out of all tested concentrations for each single allergen test solution in patients and controls was taken to generate ROC curves

The ROC curve describes how accurately the test can distinguish patients from controls. The greatest AUC values for concentration‐independent ROC curves were determined for %CD63^+^ basophils as characteristic, for ω5‐gliadins (0.908), HMW‐GS (0.867), and gluten (0.850) (Table 5; Figure 3). Concentration‐independent ROC curves were generated from the maximum values for %CD63^+^ basophils out of all tested concentrations for each single ATS in patients and controls. The optimal discrimination threshold (cut‐off) for %CD63^+^ basophils, when a basophil activation is classified as “allergen response” to a ATS, was determined for best sensitivity and specificity of concentration‐independent ROC curves (Table 5). Concentration‐dependent ROC curves showed best results at 4.00 mg/ml for ω5‐gliadins and HMW‐GS, 2.00 mg/ml for LMW‐GS and gluten as well as 0.8 mg/ml for ω1,2‐, α‐ and γ‐gliadins. More information about ROC curves and results for concentration‐dependent ROC curves are presented in the Supporting Information (Tables S1 and S2; Figure S2).

**TABLE 5:** Patient and control data from concentration‐independent ROC curves for ATSs from gluten and ω5‐, ω1,2‐, α‐, and γ‐gliadins and HMW‐GS/LMW‐GS with AUC and optimal discrimination threshold for %CD63^+^ basophils (cut‐off), when a basophil activation is classified as “allergen response” to an ATS

ATS	AUC	Cut‐off (%CD63^+^ basophils)	Sensitivity (%)	Specificity (%)
ω5‐gliadins	0.908	1.8	100	70
HMW‐GS	0.867	3.0	75	100
Gluten	0.850	2.8	75	90
α‐gliadins	0.792	3.4	67	90
ω1,2‐gliadins	0.758	2.6	67	80
γ‐gliadins	0.750	3.1	67	80
LMW‐GS	0.725	2.0	75	70

Abbreviations: ATS, allergen test solutions; AUC, area under the ROC curve; HMW‐GS, high‐molecular weight‐glutenin subunits; LMW‐GS, low‐molecular‐weight glutenin subunits; ROC, receiver operating characteristic.

## 4 DISCUSSION

In this proof‐of‐principle study we show that BAT with gluten and GPT solutions is a very promising tool to better define WDEIA. Gluten and GPT induced strong basophil activation in a dose‐dependent manner in patients at most allergen concentrations, but not in controls. This is important, because the necessity of challenge tests together with one or several cofactors makes the diagnosis of WDEIA challenging. SPT and sIgE are routine diagnostic measures to detect sensitization, but SPT and sIgE to wheat flour extracts and even sIgE to ω5‐gliadins may give negative results in WDEIA. Alternative in vitro methods to confirm the diagnosis are needed.^7^

Our results are in agreement with a study by Chinuki et al. who measured basophil CD203c expression to differentiate between classical WDEIA with IgE primarily directed against ω5‐gliadins and a new WDEIA subtype caused by hydrolyzed wheat protein (HWP_A) present in a soap in Japan. Significant enhancement of CD203c expression was observed with ω5‐gliadins in patients with classical WDEIA and with HWP_A in patients sensitized by the soap, but not vice versa.^10^ However, this study is of limited value for clinical routine, as (1) they only tested five patients in each group, but no controls, (2) used only ω5‐gliadins and HWP_A, and (3) measured CD203c, a different basophil activation marker.

CD63 and CD203c are both in use as activation markers in BAT, while CD63 is the most common one. The upregulation of CD63 is closely associated with basophil degranulation induced by allergen stimuli.^19^ Hoffmann et al. (2016) reported that the upregulation of CD203c also occurs to non‐degranulation stimuli, which is not the case for CD63.^20^ Eberlein et al. (2015) recommended the combination of CCR3 as identification marker for basophils and CD63 as activation marker for basophils in BAT and this is the setup also used in the present study.^21^

In BAT, only water‐soluble allergen solutions can be tested in patient's blood. Chinuki et al. used aqueous, ethanolic, and alkaline extractions to generate ATS for BAT, but without further protein characterization.^22^

In the present study, a well‐characterized representative gluten sample was used to isolate single GPT, α‐, γ‐, ω1,2‐, and ω5‐gliadins as well as HMW‐ and LMW‐GS. Detailed information about the basophil activation in patients to GPT were obtained.^23^ The challenge of poor solubility of gluten proteins in aqueous solutions was overcome by increasing their solubility and accessibility via partial hydrolysis with pepsin.^24^

The highly specific anti‐FcεRI mAb that imitates bridging of the receptor by an allergen has been used as a positive control in BAT for numerous years. Rubio et al. analyzed %CD63^+^ basophils and the %CD63^+^/anti‐FcεRI ratio after incubation with milk protein for prediction of the outcome of an oral challenge test. They reported a significant correlation between the %CD63^+^/anti‐FcεRI ratio and the outcome of the oral challenge test, depending on the ingested dose and reaction severity in patients with food allergy.^25^

In addition, Santos et al. found a correlation between the %CD63^+^/anti‐FcεRI ratio and the reaction severity during oral food challenge to peanuts.^26^ In disagreement, there were no correlations between BAT results (maximum %CD63^+^ basophils, %CD63^+^/anti‐FcεRI ratio), diameter of wheals and erythema in SPT, severity of symptoms (grouped comparisons I, II, III) and sIgE values (Spearman correlation test, *p* > 0.05) in our study.

Basophil activation (%CD63^+^ basophils) and %CD63^+^/anti‐FcεRI ratio were significantly higher in patients for all tested GPT and gluten in most concentrations compared to controls. Additionally, a helpful characteristic is the AUC of the dose‐response curve, which combines basophil activation and sensitivity. In our study, the AUC of dose‐response curves of ω5‐ and α‐gliadins and HMW‐GS were significantly higher in patients compared to controls.

Calculating the AUC of ROC curves gives information about the discriminability of patients from controls depending on different parameters. BAT performance differed between GPT and gluten, with only acceptable results for α‐gliadins, γ‐gliadins, LMW‐GS, and ω1,2‐gliadins, but excellent results for ω5‐gliadins (AUC ROC: 0.908), HMW‐GS (AUC ROC: 0.867) and gluten (AUC ROC: 0.850). Sensitivity and specificity of basophil activation to these substances at optimal cut‐off in WDEIA patients as compared to atopic and nonatopic control subjects were good for ω5‐gliadins (sensitivity: 100%, specificity: 70%), HMW‐GS (sensitivity: 75%, specificity: 100%), and gluten (sensitivity: 75%, specificity: 90%) respectively. The maximum %CD63^+^ basophils turned out to be the best parameter to differentiate between patients and controls, with significant differences for all tested allergens. It is conspicuous that sensitivity and specificity were higher for ω5‐gliadins, HMW‐GS and gluten than for ω1,2‐, α‐, and γ‐gliadins, and LMW‐GS, because ω5‐gliadins and HMW‐GS have previously been identified as most relevant allergens in patients with WDEIA.^1^, ^2^, ^3^, ^4^

Matsuo et al. recommended to determine sIgE against epitopes of ω5‐gliadins and HMW‐GS in combination for WDEIA diagnosis.^27^ Based on our results, we can also confirm this recommendation for their use in BAT. Other allergenic GPT were less important in our study.^2^, ^4^ BAT identified the sensitization profile of WDEIA patients to be particularly directed against ω5‐gliadins and HMW‐GS, but in individual patients also against α‐gliadins, γ‐gliadins, LMW‐GS, and ω1,2‐gliadins. For example, two patients (p6, p9) showed high responses to LMW‐GS 59.8 (p9) and 31.8 (p6) %CD63^+^ basophils, concentration 4.0 mg/ml).^1^, ^2^, ^3^, ^4^

One limitation of our study is the comparatively small number of WDEIA patients and controls. Due to the very low prevalence of WDEIA overall, a single‐center study such as ours can only include a certain number of individuals from the surrounding area. Our main intent was to identify the most suitable ATS for use in BAT from the panel of different gluten and GPT preparations tested. Now that we have identified ω5‐gliadins, HMW‐GS and gluten as most promising ATS, further work with more WDEIA patients from multiple centers is needed to put the cut‐off levels on a broader basis and include WDEIA patients with negative SPT, WDEIA patients with positive SPT, but positive challenge only with cofactors as well as individuals who are sensitized (wheat IgE‐positive), but clinically tolerant as proven by oral challenge.

According to the results of the proof‐of‐principle study we showed the potential of the BAT as alternative to routine SPT and sIgE measurements in WDEIA diagnosis. The BAT turned out to be promising to study the allergenicity of different GPTs, which becomes only possible after special preparation to increase water solubility, as required for BAT. Our findings indicate the use of %CD63^+^ basophils as best parameter to discriminate between patients and controls and highlight the allergenicity particularly of ω5‐gliadins and HMW‐GS for WDEIA.

## CONFLICT OF INTEREST

B. Eberlein received methodological and technical support from Buehlmann Laboratories AG (Schönenbuch, Switzerland). The other authors declare no competing interests.

## AUTHOR CONTRIBUTIONS

Conceptualization: Angelika Miriam Gabler, Julia Gebhard, Bernadette Eberlein, Tilo Biedermann, Katharina Anne Scherf, and Knut Brockow; Formal analysis, investigation, and methodology: Angelika Miriam Gabler and Julia Gebhard; Data curation: Angelika Miriam Gabler, Julia Gebhard and Bernadette Eberlein; Funding acquisition, resources, and Supervision: Bernadette Eberlein, Tilo Biedermann, Katharina Anne Scherf, and Knut Brockow; Visualization: Angelika Miriam Gabler; Writing—original draft: Angelika Miriam Gabler; Writing—review & editing: Julia Gebhard, Bernadette Eberlein, Tilo Biedermann, Katharina Anne Scherf, and Knut Brockow. All authors read and approved the final manuscript.

## Supporting information

Supporting Information 1
