# Supplementary material for: The basophil activation test differentiates between patients with wheat‐dependent exercise‐induced anaphylaxis and control subjects using gluten and isolated gluten protein types
Source: Clin Transl Allergy. 2021 Aug 5;11(6):e12050. doi: 10.1002/clt2.12050 (PMC8340350; doi:10.1002/clt2.12050)
Supplement: Supplementary file 1 — Supporting Information 1 [file CLT2-11-e12050-s001.docx]

**The basophil activation test differentiates between patients with wheat-dependent exercise-induced anaphylaxis and control subjects using gluten and isolated gluten protein types**

Angelika Miriam Gabler^1^, Julia Gebhard^2^ MD, Bernadette Eberlein^2^ MD, Tilo Biedermann^2^ MD, Katharina Anne Scherf^1,3, #,*^ PhD, Knut Brockow^2, #^ MD

^1^ Leibniz-Institute for Food Systems Biology at the Technical University of Munich, Freising, Germany

^2^ Department of Dermatology and Allergy Biederstein, Faculty of Medicine, Technical University of Munich, Biedersteiner Strasse 29, D-80802 Munich, Germany

^3^ Department of Bioactive and Functional Food Chemistry, Institute of Applied Biosciences, Karlsruhe Institute of Technology (KIT), Karlsruhe, Germany

^#^ Authors share co-senior authorship

**SUPPORTING INFORMATION**

**SUPPORTING METHODS**

**Preparation of BAT allergen test solutions**

**Reagents and materials**

Gluten and wheat flour were from Hermann Kröner GmbH (Ibbenbüren, Germany) and Rosenmühle GmbH (Ergolding, Germany). All reagents and chemicals were from Sigma-Aldrich (Darmstadt, Germany), Merck (Darmstadt, Germany), Honeywell (Offenbach, Germany) and J.T. Baker (Arnhem, Netherlands). Ultra-pure water was purified with an Arium 611VF water purification system (Sartorius, Goettingen, Germany). Dialysis tubes with a molecular cut-off of 12-14 kDa (Medicell International Ldt, London, England) were used for protein purification. Membrane filtration was done with filters of regenerated cellulose with a pore size of 0.45 μm (GE Health Care, Chicago, USA).

**Isolation of gluten protein types**

*Extraction of gliadin and glutenin fractions*

Gliadins and glutenins were extracted from wheat gluten according to the modified Osborne fractionation by Wieser et al.^1^ Gliadins were extracted with 60% ethanol and glutenins from the residue with a reducing 1-propanol-buffer-mixture 50/50 (v/v). The buffer consisted of 0.05 mol/L Tris/HCl, pH 7.5. Furthermore, 2 mol/L (w/v) urea, and 0.06 mol/L (w/v) dithiothreitol were added to the mixture.

*Isolation of α-, γ-, ω1,2- and ω5-gliadins*

After dialysis and lyophilization, the gliadin extract was dissolved in 60% ethanol (10 mg/mL) and α-, γ-, ω1,2- and ω5-gliadins were separated according to their characteristic retention times and collected by preparative reversed-phase high-performance liquid chromatography (RP-HPLC). Preparative RP-HPLC was performed with a HPLC-system (Jasco, Gross-Umstadt, Germany), a Jupiter C_18_ column (10 × 250 mm; 5 μm; 30 nm) and gradient elution (A: water, 0.1% trifluoroacetic acid (TFA), B: acetonitrile, 0.1% TFA) at 50 °C. The HPLC system was coupled with a fractionation collector CHF 122SC (Advantec MFS, Dublin, CA, USA). Jasco Galaxie Chromatography Data Software and Fracoll fractionation software were used. The volume of the collected fractions was reduced using rotary evaporation, and the solutions were lyophilized.^2,3^

*Precipitation of the HMW- and LMW-GS*

The HMW-GS and LMW-GS were precipitated sequentially according to Melas et al.^4^ directly from the glutenin extract Acetone was added up to percentage of 40% to the glutenin extract. The solution was mixed for 1 min and left to stand for 10 min at 22 °C to precipitate the HMW-GS. After centrifugation (3750 × *g*, 30 min, 20 °C), the precipitate containing the HMW-GS was collected. The supernatant was decanted and acetone was added to a percentage of 80%. The solution was mixed for 1 min and left to stand for 10 min at 22 °C to precipitate the LMW-GS. After centrifugation (3750 × *g*, 30 min, 20 °C), the precipitate containing the LMW-GS was collected and the supernatant discarded.

The identity and purity of the isolate gluten protein types was checked by sodium dodecyl sulfate polyacrylamide gel-electrophoresis and analytical RP-HPLC (data not shown).

**References**

1. Wieser H, Antes S, Seilmeier W, Quantitative determination of gluten protein types in wheat flour by reversed-phase high-performance liquid chromatography. J Cereal Sci 1998: 75, 644-650.

2. Schalk K, Lexhaller B, Koehler P, Scherf KA. Isolation and characterization of gluten protein types from wheat, rye, barley and oats for use as reference materials. PloS One 2017;12:e0172819.

3. Lexhaller B, Colgrave ML, Scherf KA. Characterization and relative quantitation of wheat, rye, and barley gluten protein types by liquid chromatography-tandem mass spectrometry. Front. Plant Sci 2019;10:1530.

4. Melas V, Morel MH, Autran JC, Feillet P. Simple and rapid method for purifying low molecular weight subunits of glutenin from wheat. Cereal Chem 1994:234–37.

**SUPPORTING TABLES**

**Table S1.** Area under the curve (AUC) from concentration-dependent receiver operating characteristic (ROC) curves from WDEIA patient and control data for allergen test solutions (ATS) from gluten and ω5-, ω1,2-, α-, and γ-gliadins and high- and low-molecular-weight glutenin-subunits (HMW-/LMW-GS) for %CD63^+^ basophils with corresponding concentration of ATS.

| **ATS** | **c [mg/mL]** | **AUC** |
| --- | --- | --- |
| ω5-gliadins | 4.00 | 0.975 |
|  | 2.00 | 0.650 |
|  | 0.80 | 0.783 |
|  | 0.40 | 0.658 |
|  | 0.08 | 0.767 |
| ω1,2-gliadins | 4.00 | 0.742 |
|  | 2.00 | 0.688 |
|  | 0.80 | 0.758 |
|  | 0.40 | 0.742 |
|  | 0.08 | 0.700 |
| α-gliadins | 4.00 | 0.725 |
|  | 2.00 | 0.800 |
|  | 0.80 | 0.817 |
|  | 0.40 | 0.775 |
|  | 0.08 | 0.858 |
| γ-gliadins | 4.00 | 0.704 |
|  | 2.00 | 0.733 |
|  | 0.80 | 0.767 |
|  | 0.40 | 0.742 |
|  | 0.08 | 0.567 |
| HMW-GS | 4.00 | 0.817 |
|  | 2.00 | 0.800 |
|  | 0.80 | 0.675 |
|  | 0.40 | 0.750 |
|  | 0.08 | 0.558 |
| LMW-GS | 4.00 | 0.692 |
|  | 2.00 | 0.783 |
|  | 0.80 | 0.567 |
|  | 0.40 | 0.667 |
|  | 0.08 | 0.675 |
| gluten | 4.00 | 0.742 |
|  | 2.00 | 0.750 |
|  | 0.80 | 0.733 |
|  | 0.40 | 0.742 |
|  | 0.08 | 0.600 |

**Table S2.** Patient and control data from concentration-dependent receiver operating characteristic (ROC) curves for allergen test solutions (ATS) from gluten and ω5-, ω1,2-, α-, and γ-gliadins and high- and low-molecular-weight glutenin subunits (HMW-/LMW-GS) with area under the ROC curve (AUC) and optimal discrimination threshold for %CD63+ basophils (cut-off), when a basophil activation is classified as “allergen response” to an allergen test solution (ATS).

| **ATS** | **cut-off**  **[%CD63^+^ basophils]** | **c [mg/mL]** | **sensitivity [%]** | **specificity [%]** |
| --- | --- | --- | --- | --- |
| **ω5-gliadins** | 1.4 | 4.00 | 100 | 90 |
| **ω1,2-gliadins** | 2.6 | 0.80 | 50 | 90 |
| **α-gliadins** | 2.8 | 0.80 | 67 | 100 |
| **γ-gliadins** | 4.4 | 0.80 | 25 | 100 |
| **HMW-GS** | 2.2 | 4.00 | 75 | 90 |
| **LMW-GS** | 2.3 | 2.00 | 67 | 90 |
| **gluten** | 2.5 | 2.00 | 50 | 90 |

**SUPPORTING FIGURES**


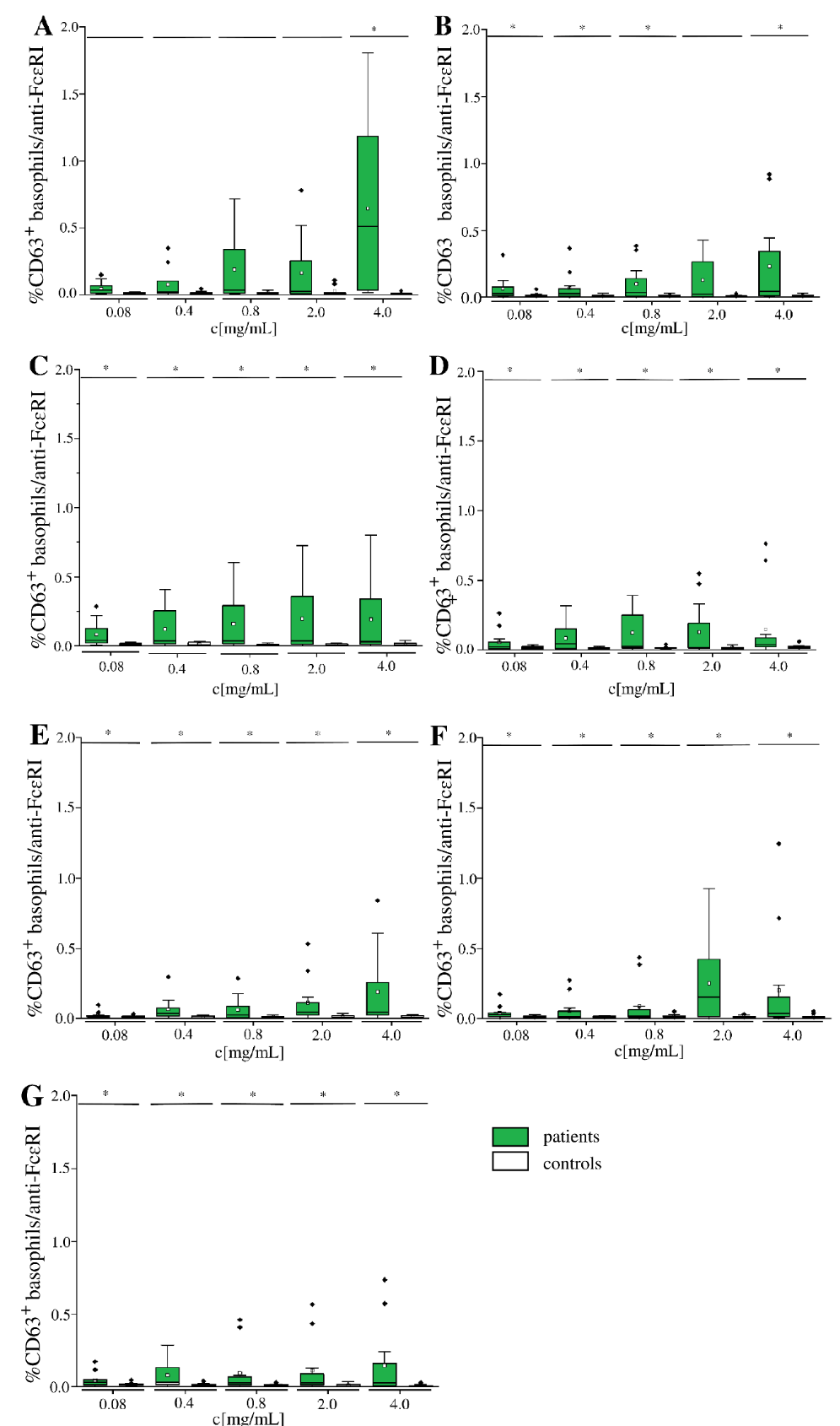


**Figure S1.** Dose-dependent %CD63^+^ basophils/anti-FcɛRI ratio in patients with WDEIA (green) and controls (white) using allergen test solutions from ω5-gliadins (A), ω1,2-gliadins (B), α-gliadins (C), γ-gliadins (D) high- (HMW-GS, E) and low-molecular-weight glutenin subunits (LMW-GS, F) and gluten (G) at concentrations of 4.0, 2.0, 0.8, 0.4 and 0.08 mg/mL. Significant differences between patients and controls are indicated by asterisks (one-way ANOVA, Dunn’s post hoc test, p<0.001). Diamonds indicate individual outliers of greater than the third quartile or less than the first quartile.


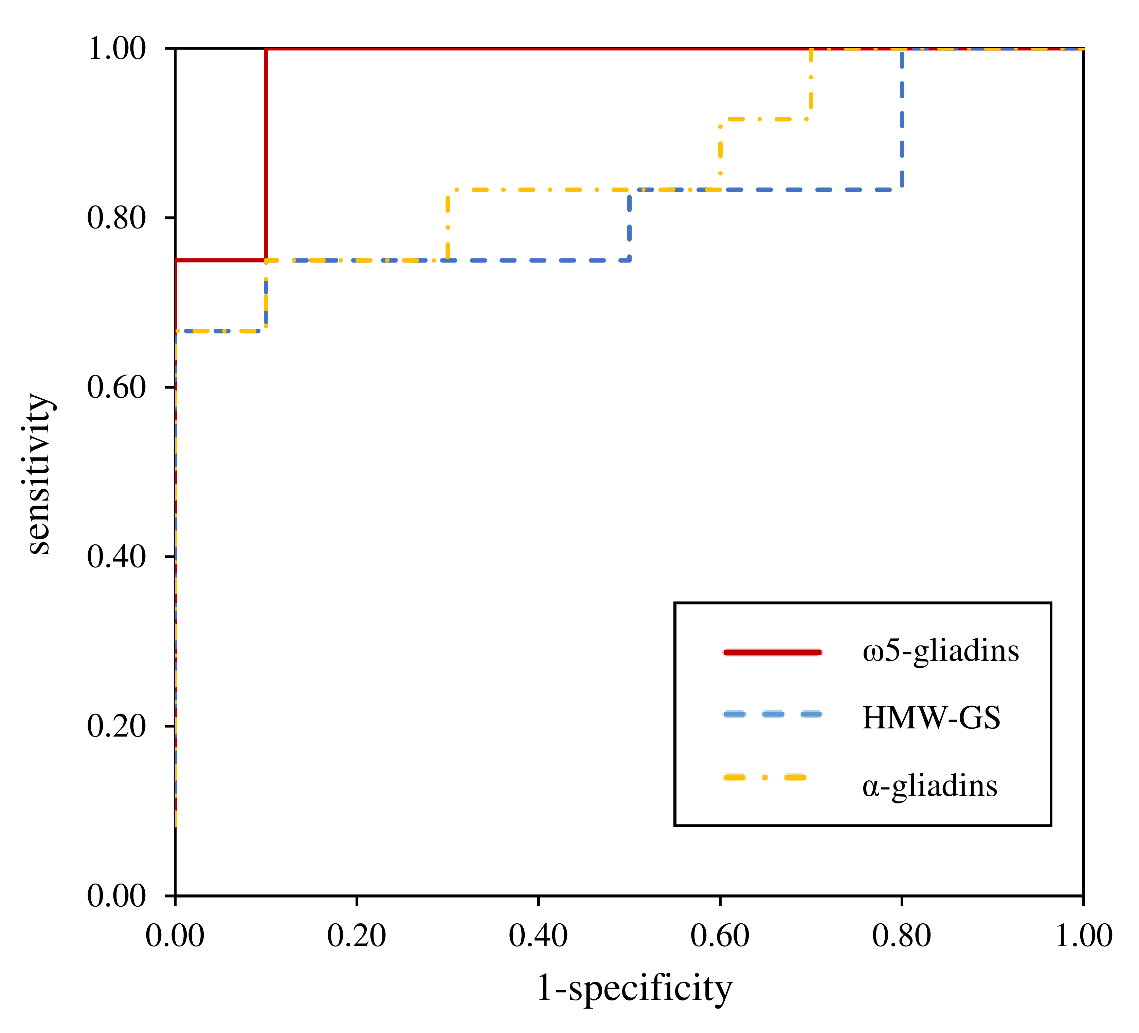


**Figure S2.** Concentration-dependent receiver operating characteristic (ROC) curve for ω5-gliadins, high-molecular-weight glutenin subunits (HMW-GS) and α-gliadins, which showed the best results for sensitivity and specificity. The concentration of 4.00 mg/mL showed the best results (highest area under ROC curve) for ω5-gliadins and HMW-GS, and that of 0.08 mg/mL for α-gliadins.
